# Effectiveness of Wearable Technologies in Supporting Physical Activity and Metabolic Health in Adults with Type 2 Diabetes: A Systematic–Narrative Hybrid Review

**DOI:** 10.3390/healthcare13192422

**Published:** 2025-09-24

**Authors:** Alessandra Laffi, Michela Persiani, Alessandro Piras, Andrea Meoni, Milena Raffi

**Affiliations:** 1 Department of Biomedical and Neuromotor Sciences, University of Bologna, 40126 Bologna, Italy; alessandra.laffi3@unibo.it (A.L.); michela.persiani3@unibo.it (M.P.); andrea.meoni@unibo.it (A.M.); 2 Department of Quality of Life Studies, University of Bologna, 47921 Rimini, Italy; alessandro.piras3@unibo.it

**Keywords:** type 2 diabetes mellitus, systematic–narrative hybrid review, wearable devices, physical activity, technological support

## Abstract

**Background**: Physical activity is essential in the prevention and management of type 2 diabetes mellitus (T2D), yet adherence to recommended activity levels remains insufficient. Wearable electronic devices have emerged as tools to support physical activity through self-monitoring and enhanced user engagement. This review synthesizes current evidence on the effectiveness of wearable technologies in improving adherence to physical activity and promoting clinical and metabolic health in adults with T2D. **Methods**: The review was conducted using systematic search strategies in PubMed and Scopus. We included studies that involved the use of wearable devices to monitor physical activity for at least seven consecutive days. The reported outcomes were related to physical activity adherence or clinical–metabolic health. Thirty-two studies met the inclusion criteria and were analyzed in terms of study design, device type, intervention characteristics, and outcomes. **Results**: Wearable devices were used either for monitoring daily activity in free-living conditions or within structured, often supervised, interventions. Most studies reported increased physical activity, particularly in step count. Several studies showed improvements in blood pressure and lipid profile, while results for HbA1c and BMI were mixed. Structured interventions with behavioural support produced more consistent and clinically relevant outcomes than passive monitoring alone. **Conclusions:** Wearable technologies can support physical activity in adults with T2D, especially when integrated into structured behavioural programmes. From a clinical standpoint, they may serve as useful tools to enhance lifestyle adherence, particularly when combined with professional support. Their inclusion in care pathways could help personalize interventions and improve long-term self-management.

## 1. Introduction

Type 2 diabetes mellitus (T2D) is an escalating global health crisis. As of 2022, an estimated 828 million adults worldwide were living with diabetes, representing an increase of 630 million cases since 1990, and reflecting an age-standardized global prevalence of 13.9% in women and 14.3% in men [1]. T2D requires lifelong clinical management, and lifestyle interventions, particularly those promoting sustained engagement in physical activity, are fundamental to improving metabolic outcomes and preventing complications.

Physical activity improves insulin sensitivity, facilitates glucose uptake in skeletal muscle and reduces visceral adiposity, thereby enhancing glycaemic control, often reflected in lower HbA1c levels. It also supports weight regulation and reduces cardiovascular risk factors such as hypertension and dyslipidaemia. When implemented together with dietary modifications and pharmacotherapy, physical activity serves as a cornerstone of comprehensive diabetes care, helping individuals with T2D to achieve and maintain key metabolic targets [2,3,4].

Despite the robust amount of evidence supporting the benefits of regular physical activity as a therapeutic measure for T2D patients is well known and accepted, adherence to exercise recommendations remains suboptimal in both at-risk populations and those already diagnosed with the disease. A substantial proportion of people fail in meeting the minimum recommendation of 150 min per week of moderate to vigorous aerobic exercise [5].

This lack of compliance can be attributed to a range of barriers occurring at multiple levels, including factors related to patients, healthcare providers, and the healthcare system itself. At the patient level, common obstacles include low motivation, fear of experiencing hypoglycaemic episodes, lack of confidence or knowledge about safe exercise practices, comorbid conditions and perceived lack of time or energy. In many cases, patients also report frustration regarding the complexity of managing insulin therapy and nutrition in combination with exercise, as well as concerns about safety and performance limitations during exercise [5,6]. Equally important are the structural and professional-level barriers that hinder effective physical activity promotion. At the practitioner level, exercise is often not systematically incorporated as a structured component of diabetes management. When offered, recommendations tend to be generic and insufficiently tailored in terms of type, intensity, duration, and progression, thereby limiting their therapeutic effectiveness and long-term adherence. Furthermore, the absence of coordinated care models and the limited availability of trained personnel or dedicated exercise facilities contribute to the difficulty in translating physical activity guidelines into sustainable patient routines [3,6].

In order to deliver effective physical activity support to individuals with diabetes, there is an urgent need for the development of clinically effective, cost-efficient, and scalable interventions that can translate supervised physical activity advice into sustained self-managed activity [7].

In recent years, wearable activity-tracking technologies, including pedometers, accelerometers, fitness trackers, smartwatches, and smartphone-based applications, have garnered growing attention as tools to encourage physical activity and support self-management in people living with chronic conditions such as T2D. These devices offer functionalities like real-time feedback, step counting, heart rate monitoring, and behaviour-based prompts to foster sustained engagement in physical activity. Some studies have also implemented more advanced, multiparametric systems that integrate blood pressure monitors, glucometers, and apps for comprehensive lifestyle tracking; owing to their accessibility and relative affordability, these technologies represent promising complements to traditional care [8,9,10]. From a clinical standpoint, wearable technologies have the potential to bridge a critical gap between lifestyle recommendations and long-term behavioural adherence. Their integration into routine care could support more personalized counselling, enable remote monitoring, and facilitate timely adjustments to treatment plans based on real-time activity data. In particular, these tools may empower nurses, case managers, and other non-physician providers to deliver structured physical activity interventions with greater continuity and scalability, especially in settings with limited access to exercise specialists or structured programmes [11]. However, despite their increasing use in clinical and real-world settings, the actual impact of these diverse technological tools on adherence to physical activity and on key metabolic outcomes in adults with T2D is still not fully elucidated. This narrative review primarily aims to explore and synthesize the current evidence regarding the effectiveness of wearable and digital devices in promoting physical activity and improving clinical–metabolic health in adults with T2D. As a secondary objective, this review discusses, within the perspective for clinical practice, the potential integration of wearable technologies into multidisciplinary, nurse-led, and digitally supported care models for T2D management.

## 2. Materials and Methods

A narrative review of the scientific literature was conducted to explore the effectiveness of wearable electronic devices in promoting physical activity and adherence among older adults diagnosed with T2D.

Although the review is narrative in nature, a systematic approach was adopted in the literature search and screening and selection processes to improve the transparency and reproducibility of the results. Distinct search strategies were employed for each database.

In PubMed, the following search string was used: (“wearable electronic devices” OR “fitness tracker” OR smartwatch OR pedometer OR accelerometer) AND (“physical activity” OR “exercise” OR adherence OR “self-monitoring”) AND (elderly OR “older adults” OR ageing OR aged) AND (diabetes OR “type 2 diabetes”).

In Scopus, the search was performed using the TITLE-ABS-KEY field with the following terms: TITLE-ABS-KEY (“wearable electronic devices” OR “fitness tracker” OR smartwatch OR pedometer OR accelerometer) AND TITLE-ABS-KEY (“physical activity” OR exercise OR adherence OR “self-monitoring”) AND TITLE-ABS-KEY (elderly OR “older adults” OR ageing OR aged) AND TITLE-ABS-KEY (diabetes OR “type 2 diabetes”).

The time range of the publications considered was 10 years, from 2015 to 2025, selected to capture the most recent and relevant scientific contributions in the field. Studies were included if they (i) involved adults (>18 years old) of any gender diagnosed with T2D; (ii) employed wearable electronic devices specifically for monitoring physical activity; (iii) directly assessed participants’ adherence to physical activity; (iv) had a minimum intervention duration of at least seven consecutive days of wearable device monitoring; and (v) were published in English.

Excluded studies were those that (i) used wearable devices for purposes not related to physical activity monitoring; (ii) did not directly evaluate adherence to physical activity or the effectiveness of the intervention in terms of clinical outcomes related to physical activity; (iii) involved participants with acute or chronic medical conditions that could prevent regular engagement in physical activity; and (iv) had an intervention duration of less than seven days. A minimum of seven days was considered appropriate, as this duration is commonly adopted in physical activity research to account for day-to-day variability and to provide a reliable representation of habitual behaviour, including differences between weekdays and weekends [10,12,13].

The selection of studies was conducted independently by three authors (A.L., M.R. and M.P.) using a double-blind screening procedure. Each investigator assessed the titles, abstracts, and full texts of potentially eligible studies without knowledge of the other’s decisions. Disagreements were resolved through consensus-based discussion in order to minimize selection bias and enhance methodological rigour. The entire screening and selection process was managed using the Rayyan QCRI web-based platform.

**Figure 1:**
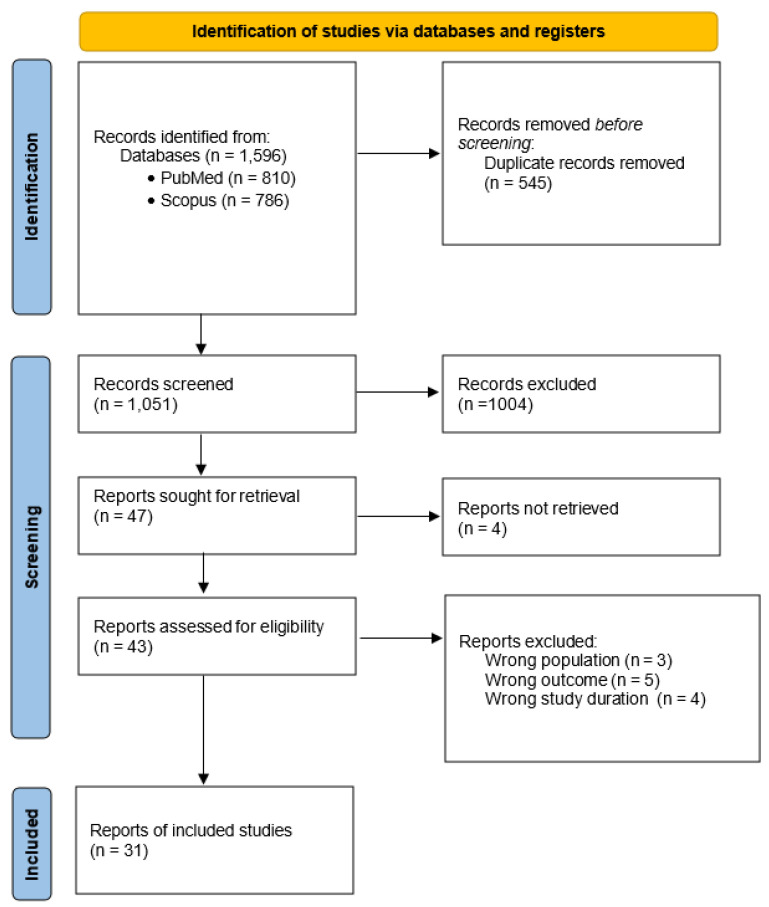
PRISMA flow diagram of the study selection.

A total of 1596 articles were initially identified through database searches. After removing 545 duplicates, 1051 records remained for title and abstract screening, of which 1004 were excluded as clearly irrelevant. Forty-seven articles were retrieved for full-text assessment; however, one study was excluded due to formal retraction, and three others could not be accessed in full text, despite repeated attempts through institutional databases and interlibrary services. As a result, 43 articles were included in the eligibility evaluation. Following detailed assessment against the predefined criteria, 12 articles were excluded: 3 due to ineligible population, 5 due to irrelevant outcomes, and 4 due to insufficient intervention duration. In total, 31 articles were included in the final review. The detailed selection process is illustrated in Figure 1.

## 3. Results

### 3.1. Study Designs and Types of Wearable Technologies Used

This narrative review includes 31 studies that investigated the effectiveness of wearable technologies in promoting physical activity and improving clinical and metabolic outcomes in adults with T2D. The majority were randomized controlled trials (n = 23), accompanied by seven observational studies, comprising four with a prospective or longitudinal design [9,10,14,15] and three that were cross-sectional [16,17,18], along with one quasi-experimental study with a pre–post design and no control group [19]. All included studies required a minimum physical activity monitoring period of seven consecutive days, consistent with the review’s eligibility criteria.

The populations studied varied widely in sample size (ranging from 19 to over 4000 participants), age (from middle-aged adults to older populations), gender distribution, and geographic setting. The studies were conducted across a diverse set of countries, including the United States, Canada, the United Kingdom, Italy, France, Spain, Nigeria, South Africa, Malaysia, Oman, Indonesia, Japan, China, Brazil, Turkey, Israel, and Cameroon. This diversity provides a comprehensive and representative perspective on how wearable technologies are being implemented across both clinical and real-world settings in the management of T2D.

The wearable devices employed to monitor physical activity across the included studies varied significantly in terms of technological complexity, interface usability, feedback capabilities, and the level of user engagement required. Some devices operated as basic step counters, offering passive data collection with no interactive features. Others incorporated more advanced functionalities, including real-time feedback, goal-setting tools, and behavioural prompts designed to support physical activity adherence. In several studies, wearable devices were embedded within broader digital health platforms, enabling integration with clinical monitoring tools or self-management applications. Based on their functional roles and technological features, the devices can be categorized into three main groups: (1) pedometers, (2) accelerometers and (3) multiparametric or integrated digital health systems.

A detailed summary of the included studies, including study design, population characteristics, device types, monitoring duration, and primary outcomes, is provided in Table 1.

#### 3.1.1. Pedometers

A pedometer is a compact device designed to record physical activity, most commonly by counting steps [20,29]. It is typically described as a wireless activity sensor that can be worn in various positions, including attached to a shoe [20], clipped to the waist [12,22,29], integrated into clothing [8,12], or worn around the neck [7]. Pedometers are generally considered low-cost tools, making them accessible for large-scale public health applications and individual self-monitoring [29]. While basic pedometers primarily measure step count [8,12,13,19,21,23,28,29,31,34,35], more advanced models may also estimate distance walked, energy expenditure [7,20,22], and activity duration [7], although the range and precision of these functions can vary depending on the device.

Some devices are capable of capturing the duration of moderate-intensity walking [7]. For example, the Omron HJ-321 Triaxis model records daily step counts, aerobic steps, walking distance, and calories burned. Aerobic steps, defined as walking at ≥60 steps per minute for over 10 min, offer an objective indicator of walking intensity and are considered clinically relevant for assessing moderate-intensity activity [22].

Overall, pedometers provide an objective measure of ambulatory physical activity. Nevertheless, certain studies have noted that these devices may primarily detect vertical movements of the lower limbs, potentially limiting their ability to capture other types of physical activity, such as upper-body motion or non-ambulatory tasks [13].

#### 3.1.2. Accelerometers

Accelerometers are essential wearable devices in the study of physical activity and sedentary behaviour. These devices record movement acceleration, enabling the quantification of physical activity and sedentary time under real-life conditions [10,24]. They provide continuous and accurate data, including the ability to detect low-intensity movements [10,17]. The accelerometers described in the included studies varied in both technical configuration and placement. Some were uniaxial accelerometers that measure vertical acceleration only [16,17,24], such as the MyWellness Key, which is typically worn on the waist [16,17,24,25,30]. Others were triaxial accelerometers capable of detecting movement across three axes [10,14,33]. These include wrist-worn devices like the Axivity AX3 [10], pocket-carried models [14], and portable ambulatory triaxial accelerometers worn on the hip or body [33]. In addition, some studies employed accelerometers integrated into smartphones [33] or commercial fitness trackers, such as Fitbit [9].

These devices operate by continuously recording raw movement signals, which are then processed using device-specific algorithms to generate meaningful metrics. For example, the MyWellness Key expresses activity levels in counts per minute [16,24], whereas the Axivity AX3 reports data in milligravities (mg) [10]. Predefined intensity thresholds were applied to classify and quantify time spent at different physical activity intensities [16,24].

The main outcomes derived from accelerometer data in studies involving adults with type 2 diabetes are listed below:

(1) Time spent at specific intensities: Most studies measured the duration of physical activity at different intensity levels: light (LPA), moderate (MPA), and vigorous (VPA), often reported collectively as moderate-to-vigorous physical activity (MVPA). The cut-points used to define these intensities varied across devices and studies, and were typically based on counts per minute or milligravity (mg) thresholds [10,16,24,25,30].
(2) Sedentary time (SED-time): This was defined as the combination of non-wear time during waking hours (excluding non-trackable activities such as swimming, typically recorded in logbooks) and periods in which the device registered activity levels below a defined threshold (e.g., <100 counts/min for the MyWellness Key, corresponding to sitting or lying down) [16,24]. Several studies reported a decrease in sedentary time after the interventions [16,24,30].
(3) Total physical activity volume was assessed using different metrics across the included studies. Some devices, such as the MyWellness Key, expressed total activity using unitless scores called MOVEs (where 2.5 MOVEs = 1 MET-minute), while others, like the Active Style Pro HJA-750C, reported activity in MET-hours per week [16,24,33].
(4) Some devices also measured daily step count and estimated energy expenditure, providing additional indicators of physical activity volume [16].

#### 3.1.3. Multiparametric or Integrated Digital Health Systems

These systems combine wearable sensors with mobile applications to monitor multiple parameters of physical activity, including frequency, intensity, duration, and progression. By integrating physiological data and providing real-time feedback, they enable standardized and objective tracking of exercise-related metrics.

In the study by Li et al. [32], a multiparametric system comprising the R Plus Health mobile application and a wearable chest strap was used to objectively assess physical activity. The system tracked time spent within the target heart rate zone, provided real-time feedback, and automatically calculated the cumulative duration at the prescribed intensity, offering a standardized measure of both exercise intensity and duration.

### 3.2. Physical Activity Promotion: Passive Monitoring vs. Structured Interventions

In this narrative review, all included studies were categorized into groups according to predefined criteria, based on the implementation design of the physical activity interventions.

Physical activity interventions were considered structured only if they included clearly defined and quantifiable targets in terms of duration and/or intensity (e.g., weekly minutes, daily step counts, METs, or percentage of maximum heart rate), or if they incorporated exercise sessions with a clearly specified content, frequency, duration, and modality, regardless of whether the sessions were supervised. Interventions that only provided general World Health Organization (WHO) or American Diabetes Association (ADA) physical activity guidelines, without personalized or monitored targets, as well as those that encouraged general increases in physical activity levels were excluded from this category. Based on these criteria, the interventions were classified into three categories: structured and supervised (n = 7), structured but unsupervised (n = 15), and unstructured and/or monitoring-based interventions (n = 9).

This categorization underscores the heterogeneity in intervention design across the included studies. The following sections present the main findings by group, aiming to elucidate how varying degrees of structure and supervision may influence physical activity behaviours and clinical outcomes, both within and across the intervention categories.

#### 3.2.1. Exercise-Related Outcomes in Structured and Supervised Interventions

Structured and supervised interventions consisted of exercise sessions conducted under the direct guidance of qualified personnel, such as physiotherapists, exercise trainers, or healthcare professionals. Findings from multiple clinical trials indicated that this approach, typically incorporating aerobic and/or resistance training, consistently yielded broadly consistent health benefits in individuals with type 2 diabetes.

Participants generally experienced improved glycemic control, with significant reductions in HbA_1_c compared to standard care, observed both in the short- and long-term interventions. Cardiorespiratory fitness also improved, as indicated by increases in VO_2_max and better performance on functional walk tests such as the six-minute walk test [16,24,25,27]. Muscular strength increased, particularly in protocols including resistance training, and in several cases, these improvements were significantly greater in intervention groups than in controls. Modest reductions in body weight, BMI, and waist circumference were commonly reported, indicating improved body composition, including increased lean mass and reduced visceral fat. While significant between-group differences were sometimes observed in the short term, these were not always maintained over time, with improvements generally aligning with greater increases in physical activity and reductions in sedentary behaviour [22,30].

These interventions also led to a more favourable metabolic profile, with reductions in LDL cholesterol and triglycerides and improvements in fasting glucose, as well as attenuated systemic inflammation evidenced by decreases in inflammatory markers like high-sensitivity C-reactive protein [24,25,30]. In addition, several interventions resulted in improved liver function, including reductions in hepatic enzyme levels (ALT, γ-GT), as well as better scores on indices of non-alcoholic fatty liver disease (NAFLD), suggesting a decrease in ectopic liver fat [22,37]. Overall, despite minor variability in the magnitude of changes across individual studies, the pattern of results is strikingly consistent: supervised exercise confers multi-faceted improvements in glycemic control, fitness, anthropometric measures, cardiovascular risk factors, inflammation, and liver health in people with type 2 diabetes [16,24,25,27].

The magnitude of clinical and behavioural effects differs across structured, but self-directed physical-activity programmes and these differences align with the intensity and frequency of remote support offered to participants. The highest-support trials employ continuous or near-continuous monitoring technologies and provide frequent, highly personalized feedback drawn from real-time data [7,8,20,29,32,38]. Trials providing a moderate level of support combine daily self-monitoring with regularly scheduled touchpoints such as structured educational sessions, one-to-one counselling, or peer-support contacts delivered at predefined intervals [12,19,21,31,35]. Lastly, interventions offering only limited support provide participants with an activity-tracking device and an initial goal; subsequent contacts are infrequent and primarily intended for data collection, with no structured counselling or regular feedback [18,28,34,36].

#### 3.2.2. Exercise-Related Outcomes in Structured but Unsupervised Interventions

In structured but unsupervised interventions, participants performed the prescribed physical activity independently, without direct supervision. However, the interventions maintained a high level of structure, incorporating clearly defined targets, feedback systems, and digital platforms to support adherence and enable continuous remote interaction between participants and healthcare or research personnel.

Significant increases in the amount of physical activity compared with control groups were observed in interventions involving moderate to high levels of support, with each study emphasizing distinct elements that contributed to their effectiveness.

In the study by Agboola et al., the text-message intervention led to increased walking in the intermediate follow-up period, highlighting the importance of sustained engagement strategies [20]. Gu et al. demonstrated that physical activity increased more substantially when a pedometer was combined with home blood pressure monitoring, suggesting a synergistic effect between these two components [29]. Kooiman et al. demonstrated that combining a web-based application with an activity tracker encouraged more frequent engagement in moderate-to-vigorous physical activity [8]. The study by Li et al., which implemented an mHealth application in combination with a heart-rate strap, indicated that remotely guided exercise may be more effective in promoting meaningful physical activity than self-reported routines, even when the total duration of activity is lower [32]. Yom-Tov et al., showed that SMS messages generated through reinforcement learning significantly increased both the amount and speed of walking, underscoring the impact of highly personalized communication [38]. Additionally, studies by Alghafri et al., Johnson et al., and L.P. de Oliveira et al. found that incorporating step-tracking into educational or dietary programmes reduced sedentary behaviour and supported more consistent daily physical activity [12,21,31]. Sazlina et al. further demonstrated that combining peer support with personalized feedback enhanced participation in moderate-intensity exercise [35]. Overall, these findings suggest that improvements in physical activity are greater and more sustained when the support provided is personalized, interactive, and continuous.

Across fifteen publications in this category, evidence for the impact of step-based physical-activity interventions on glycated hemoglobin (HbA1c) remains mixed and context-dependent. Eight studies reported significant reductions in HbA1c within the intervention groups; however, only five demonstrated a statistically significant difference when compared to their respective control groups [7,18,28,34,38]. Notably, neither increased technological sophistication nor greater intensity of support translated into superior glycaemic outcomes. Interventions incorporating bidirectional messaging or web-based platforms did not demonstrate greater efficacy than usual care, and the initially favourable effect observed in Miyauchi et al. was no longer evident at the six-month follow-up [7,8,20,32]. Similarly, programmes focused on periodic counselling sessions were effective in increasing ambulatory activity but did not produce significant changes in HbA1c compared to control conditions [12,21,31,35]. This may be due to the absence of a clearly defined behavioural target, such as a specific daily step goal. Notably, the most consistent improvements in HbA1c among more active participants were observed in interventions that included such a quantifiable objective [18,28,34].

Multiple sources confirm that the effectiveness of step-based interventions in reducing HbA1c depends on the interplay of several factors. A higher baseline HbA1c is consistently associated with greater potential for clinically meaningful improvement. Individuals with initial values above 8.5% tend to show more pronounced reductions than those whose glycaemic control is already within target (e.g., HbA1c ≈ 6.8%). In individuals with good baseline glycaemic control, HbA1c improvements may be more likely when increases in physical activity are substantial and accompanied by greater intensity [28]. Moreover, an intervention duration of at least 10 to 13 weeks appears necessary to produce consistent effects, while shorter or markedly longer approaches have yielded mixed results.

Outcomes are also closely linked to adherence, with greater improvements observed among participants who consistently engage with the programme. In this context, objectively monitoring physical activity through wearable devices or mobile applications is more accurate and effective than relying on self-reported behaviour.

When blood-pressure outcomes are examined, reductions are observed only when the walking intervention incorporates a BP-specific component. A substantial decline in both systolic and diastolic pressure over 12 months was recorded when home BP monitoring accompanied pedometer use [29]. Likewise, a multimodal programme that combined dietary counselling, pedometer feedback, and structured WhatsApp messaging maintained the reduction for up to one year [21]. By contrast, providing a pedometer within dietary supervision produced only a modest, diastolic-limited benefit, evident solely after adjustment for baseline values [12]. Interventions focused almost exclusively on step targets remained neutral with respect to BP, despite improving other metabolic markers [28,31,34,35]. In summary, increased walking lowers blood pressure only when paired with objective BP monitoring or structured cardiovascular counselling; step goals alone do not yield a clinically meaningful effect.

Evidence for lipid modulation among structured yet unsupervised programmes is limited. A single multicomponent programme combining pedometer-guided activity with structured dietary counselling, was associated with a modest but significant reduction in triglyceride levels relative to the control group [21].

Structured lifestyle trials that include regular coaching or digital support often led to modest drops in body weight and BMI within the intervention arm, but these changes rarely reached statistical significance when compared with usual-care controls [8,12,20,21,31]. Programmes offering only minimal support, such as simple step-count targets, typically show little to no impact on these markers [28,34]. Interestingly, the handful of studies that assessed body composition objectively (e.g., percentage body fat) sometimes detected meaningful fat-mass reductions even when BMI remained unchanged, particularly when advanced remote monitoring devices and personalized feedback or peer support were used [32,35]. Still, several other trials that combined objective measurements with lighter support failed to replicate these benefits [12,34], suggesting that both the specific design of the intervention and the intensity of follow-up are critical determinants of success.

Additionally, programmes incorporating counselling or real-time feedback showed broader benefits, including reduced medication use and improved functional capacity, such as endurance, gait speed, and walking performance [19,29,32,35].

#### 3.2.3. Exercise-Related Outcomes in Unstructured and Monitoring-Based Interventions

The final category of studies discussed in this review includes interventions that did not include a standardized physical activity prescription or a predefined session structure. Instead, such studies aimed to promote autonomous increases in daily activity through motivational, educational, or technological strategies, such as feedback messages or general counselling, or, in observational research, relied entirely on passive monitoring without the implementation of an active behavioural component.

A subset of interventions explicitly reported a net increase in daily steps or minutes of activity following the intervention [13,23], offering a clear estimate of behavioural change over time. In contrast, other studies focused on average levels of activity during the study period rather than on pre–post differences [14,17], or assessed whether participants met predefined physical activity thresholds without reporting absolute increases [26]. A few further studies centred primarily on device adherence or day-level associations with metabolic outcomes, without aiming to capture changes in overall physical activity volume [9,15].

Based on these observations, the clinical outcomes reported across the nine studies in this category reveal a heterogeneous picture, particularly in relation to glycaemic control, with several interventions associated with improvements in glycaemic parameters, although the magnitude and consistency of effects varied. Some studies reported reductions in HbA1c, fasting glucose, or 2 h post-load glucose over time, even in the absence of statistically significant group-by-time interactions, suggesting that increased daily activity may contribute to better metabolic control [13,23]. In other cases, behaviourally focused approaches like motivational interviewing, which encouraged adherence to official physical activity recommendations, led to greater reductions in serum glucose, though effects on HbA1c were less pronounced [26]. Observational designs offered complementary insights: lower objectively measured daily step counts over six months were strongly associated with poorer glycaemic control [14], while intermediate levels of daily activity showed the greatest HbA1c reduction in a stratified analysis [17].

Glycaemic variability appeared reduced on days marked by at least one hour of moderate-intensity activity, based on day-level analyses [15] and adherence to digital monitoring tools appeared to correlate with more favourable baseline and follow-up HbA1c values, though not with change over time [9]. Collectively, these findings suggest that both absolute and daily activity patterns, as well as monitoring behaviours, may be relevant for glycaemic management even in the absence of formal exercise prescriptions.

Blood pressure outcomes across the studies in this group were generally modest. Most interventions did not produce significant changes in systolic or diastolic values, particularly when baseline blood pressure was already within the normal range [23,26]. A small reduction in systolic pressure was observed among participants with higher physical activity levels [17], though diastolic values remained unchanged. The most notable effects emerged in the study combining DASH (Dietary Approach to Stop Hypertension) diet and walking, which reported marked improvements in ambulatory blood pressure monitoring further supporting the intervention’s cardiovascular benefits [13].

Body composition outcomes were generally neutral across the studies, with few interventions showing significant between-group effects. No meaningful changes in BMI or other anthropometric indicators were observed in most trials [13,14,23,33]. A small but statistically significant reduction in BMI was reported in the motivational interviewing group compared to usual care [26]. One study found a favourable shift in the ratio of visceral to total adipose tissue in participants with higher physical activity levels, though overall fat and lean mass remained unchanged [17].

Cardiovascular risk markers and mortality-related outcomes were explored in a subset of studies. One large-scale observational analysis found that higher levels of daily physical activity were associated with lower all-cause, cardiovascular, and cancer-related mortality, with clear dose–response relationships observed [10]. Another study reported that increased activity levels were inversely associated with oxidative stress markers and were linked to improvements in endothelial function and lipid oxidation parameters [17]. Although not directly assessing clinical outcomes, other investigations suggested that enhanced daily activity may contribute to long-term reductions in T2D complications and cardiovascular mortality risk [23].

#### 3.2.4. Influence of Device Sophistication on Intervention Outcomes

Although the reviewed studies employed a wide range of physical activity monitoring tools, the level of technological sophistication alone does not appear to be the primary driver of clinical efficacy. Rather, the effectiveness of a device seems to depend largely on how it is integrated into the broader structure of the intervention. As noted by Timurtas et al., no significant differences in HbA1c outcomes were observed between participants in supervised exercise programmes and those using mobile applications or smartwatches. This finding suggests that advanced devices, when appropriately implemented, may achieve comparable clinical outcomes to traditional supervised models [37]. Importantly, more sophisticated devices such as accelerometers and smartwatches provide objective, high-resolution data on activity intensity, frequency, and sedentary behaviour. These capabilities enable tailored feedback, adaptive goal setting, and integration into behavioural change frameworks, potentially enhancing user engagement and adherence. Therefore, the clinical impact of wearable technologies is maximized not merely by their features, but by their integration within structured, theory-based, and feedback-driven interventions. In summary, while the presence of wearable technology is not inherently sufficient to improve outcomes, the combination of advanced monitoring capabilities with structured behavioural support appears to offer the greatest potential for improving glycaemic control and other key clinical parameters in individuals with type 2 diabetes.

### 3.3. Study Withdrawal and Retention Trends

The analysis of dropout rates across the reviewed studies reveals substantial variability, largely influenced by differences in intervention design.

Structured physical activity delivered under direct supervision was generally associated with better retention. The IDES_2 trials and the study by Alonso-Dominguez et al. showed favourable adherence, with most withdrawals attributed to illness, relocation, or limited availability [16,22,24,25,30]. Timurtas et al. reported moderate attrition, while dropout details were not clearly specified by Dahjio et al. [27,37].

Overall, supervision appears to enhance adherence by promoting accountability, structured engagement, and social support, though personal and logistical barriers remain significant obstacles to sustained participation.

Dropout rates tended to be higher in studies involving structured but unsupervised interventions, where technological issues and lack of sustained motivation were common contributing factors. Several studies reported withdrawals due to technical issues, including software malfunctions, connectivity problems, or failures in data transmission [20,32]. In some cases, discomfort with monitoring devices and joint pain, together with loss of interest and difficulty sustaining motivation, further compromised adherence [21,28].

Sociodemographic and clinical factors were occasionally associated with higher attrition, with participants who discontinued often being younger, having higher BMI, lower baseline fitness or self-efficacy, and more frequently being women [31,35]. Additional studies confirmed variability in retention, attributing dropout to adverse events, loss to follow-up, or unspecified causes [18,32,36].

Conversely, some studies reported minimal attrition, with only isolated dropouts or limited data loss [8,29,34]. In a few cases, dropout was either not clearly reported or attributed to external factors, such as the COVID-19 pandemic [12,19]. In summary, structured interventions without supervision tend to be more vulnerable to disengagement, largely due to technical barriers, limited support, and reliance on participants’ intrinsic motivation.

## 4. Discussion

This review provides a structured overview of the role wearable devices may play in the promotion of physical activity among individuals with type 2 diabetes. Their effectiveness appears to depend largely on how they are integrated into the overall intervention, particularly in relation to the level of structure, the consistency and nature of behavioural support, and the presence of clearly defined, measurable objectives [17,20,21,22,23,26,28]. Rather than serving as stand-alone interventions, wearable devices are most effective when employed as complementary components within a coherent behavioural framework. When aligned with structured guidance, regular monitoring, and sustained feedback, these technologies can enhance engagement with physical activity and contribute meaningfully to long-term self-management [12,31,34,35].

In supervised exercise interventions, where professional oversight and structured routines are central, wearable devices often play a supportive rather than a driving role. The efficacy of these interventions is largely attributed to direct human interaction, social accountability, and structured scheduling, which together create a robust motivational and behavioural framework. Within this setting, wearable devices are primarily employed to facilitate self-monitoring and progress tracking, reinforcing adherence and enabling participants to visualize their improvements over time. However, the behavioural scaffolding remains largely shaped by interpersonal support [16,24,25,27]. While such models are associated with high levels of adherence and retention, personal and logistical barriers (e.g., illness, transportation, competing responsibilities) may still limit long-term participation. Moreover, the high resource demands of supervised programmes raise important concerns regarding their scalability, economic sustainability, and accessibility, particularly within healthcare systems with limited resources or among socioeconomically disadvantaged populations [37].

Structured but unsupervised interventions, by contrast, represent a more relevant domain for wearable technologies to demonstrate their potential. In these programmes, devices often play a key role in enabling remote interaction, providing behavioural feedback, and helping participants monitor their progress toward specific goals. The review shows that wearables are most effective when combined with regular prompts, personalized feedback, and adaptive communication strategies: features that help maintain motivation and reinforce habits [8,20,21,32,38].

Results indicate that increased physical activity alone, even when facilitated through wearable use, does not consistently translate into improved glycaemic outcomes. Interventions lacking precise behavioural targets or sufficient intensity often have limited impact on HbA_1_c, suggesting that goal clarity and intervention design are as important as the technology itself. Wearables appear most effective when aligned with structured prescriptions (e.g., step goals, intensity thresholds) and when the feedback loop reinforces these targets in real time. In the absence of such structure, the device’s ability to support sustained clinical improvement may be significantly reduced [8,12,20,31,32,35].

Unstructured or passively monitored approaches, including observational studies and minimally guided interventions, further highlight the importance of intentional design [10,15,17]. While some of these programmes demonstrated modest increases in activity levels or positive metabolic associations, the results were inconsistent and often context-dependent [15,17,23]. In such cases, wearable devices functioned primarily as tools for awareness and data collection, rather than as drivers of behavioural change [9,13,23,26,33]. While they may still hold value in preventive contexts or in supporting the maintenance of baseline activity among already active individuals, their ability to produce meaningful clinical improvements appears limited in the absence of structured behavioural guidance [26,33].

Adherence and retention trends across studies offer deeper insight into the determinants of intervention effectiveness. Supervised programmes were generally associated with higher retention, likely due to the structured support they offer. In contrast, higher dropout rates were more frequently observed in unsupervised interventions that relied heavily on technology. The primary reasons for disengagement included technical difficulties (e.g., connectivity issues or device malfunction), reduced motivation over time, sociodemographic vulnerability and discomfort or unfamiliarity with the monitoring devices. These findings reinforce the need for inclusive, accessible, and user-friendly designs that support a wide range of users and accommodate different levels of digital confidence [9,26]. Collectively, these observations indicate that the clinical value of wearables depends on their integration within structured, human-supported delivery models. Accordingly, the subsequent subsection examines nurse-led and digitally enabled care pathways that implement these mechanisms in routine practice.

### 4.1. Perspectives for Clinical Practice

Within contemporary T2D management, nursing assumes a central role when combined with Lifestyle Medicine principles and the digitalisation of care [11,39,40,41]. Lifestyle Medicine offers a multidisciplinary, evidence-based framework that targets modifiable behavioural determinants. Applied through case management, it enables coordinated team care and structured self-management support. This approach mirrors the mechanisms highlighted in our review, namely clear goals, continuous feedback and timely adjustments, and helps convert monitoring into action [11,39,41].

Compared with usual care, nurse led models, characterized by structured education, proactive follow-up, and protocol-driven treatment adjustments, consistently produce modest but clinically relevant improvements in intermediate outcomes. Benefits are observed for HbA1c, systolic blood pressure, and BMI, alongside stronger self-care behaviours. These models are particularly useful in underserved and rural settings, where they mitigate access barriers and redistribute workload without loss of quality. Typical components include dietary counselling, symptom management, lifestyle-change support, psychological reinforcement, and education for diabetes self-management [11,40,41].

A concrete extension of nurse led care is the Lifestyle Medicine Case Manager Nurse (LMCMN). The LMCMN coordinates the principal pillars of lifestyle intervention, including physical activity, nutrition, sleep, stress management, tobacco exposure and social connection, and integrates objective data from wearable sensors such as steps, moderate to vigorous physical activity and heart rate. Where available, continuous glucose monitoring with time in range and home blood pressure monitoring are incorporated [11,40,41]. These data are used to set specific, measurable, achievable, relevant and time bound (SMART) goals, guide staged progression and prompt timely escalation to other professionals (primary care or diabetology, pharmacy, dietetics, physiotherapy) when targets are not achieved [40]. Recent frameworks delineate scope, competencies and role descriptors for chronic disease management, including motivational interviewing, exercise prescription protocols and digital literacy support. In Italy, Family and Community Nurses are increasingly positioned to undertake these functions as coordinators of proximity care and facilitators of digital health technologies [40,42].

Evidence for nurse led, digitally enabled programmes, including telemonitoring, app supported education for diabetes self-management and structured messaging, shows feasibility, acceptability and reductions in HbA1c compared with usual care. Higher contact intensity and tailored content are associated with larger effects. Asynchronous communication, such as secure messaging, text messages or email, may outperform synchronous calls by enabling near real time prompts at lower logistical cost, provided messages are personalized, behaviour specific and linked to measurable activity or glycaemic targets [11,41]. Across trials, engagement and satisfaction are high, attrition is commonly less than 20 per cent, and improvements in self-care behaviours are consistent. Despite favourable signals, several limitations warrant attention: interventions vary in content, intensity and duration; effects from tightly controlled trials may not generalize to routine services; and scaling is difficult because high contact models require substantial staff time, training and robust digital infrastructure [11,40,41].

From a service design perspective, a pragmatic pathway is to embed wearables within nurse led case management. Core steps include baseline triage (HbA1c, comorbidities and fall risk, digital readiness); SMART activity prescriptions (for example, plus 2000 steps per day or at least 150 min per week of moderate to vigorous physical activity, with heart rate zoned progression for deconditioned patients); brief weekly check ins for 12 to 16 weeks with tapering; explicit safety protocols (hypoglycaemia prevention, sick day rules, medication review around exercise); and predefined escalation triggers (persistent out of target HbA1c or time in range, rising blood pressure, sustained declines in activity) [11,40,41,42]. Equity can be supported through loaner devices, low literacy multilingual materials and electronic health record integrated dashboards that streamline population management and documentation. This structure preserves the strengths identified in our review, namely clear targets, feedback and continuity, while offering a scalable template for routine care [11,39,42].

### 4.2. Strengths and Limitations of This Review

This review synthesizes recent studies from multiple countries and settings and brings together both randomized and observational evidence. Clear eligibility criteria, a pragmatic classification by intervention structure, level of supervision and device type, and the requirement for at least seven consecutive days of monitoring help make comparisons meaningful and link the findings to clinical practice. Nonetheless, the included studies differ in design, device characteristics, intensity thresholds, outcome definitions and follow up duration, which limits direct comparability. Inclusion was restricted to English language publications and a small number of full texts were unavailable. In addition, rapid evolution of technologies, frequent co interventions and differences in device placement or algorithms may complicate attribution of effects to the wearable component alone. These considerations do not change the overall message of the review but should be kept in mind when applying the findings in routine care.

## 5. Conclusions

Wearables support physical activity in T2D, but their clinical impact depends on how they are embedded within structured interventions rather than on device sophistication per se. The greatest benefits occur when programmes set clear goals, provide regular monitoring and personalized feedback, and reinforce behaviour change; effects are limited when devices are used in isolation or in loosely organized contexts. Nurse-led, multidisciplinary and digitally enabled care pathways, including Lifestyle Medicine case management, offer a pragmatic route to translate monitoring into action and improve intermediate outcomes. Implementation should prioritize interoperability, safety protocols and equity enablers (device loan schemes and plain-language, multilingual materials). Future research should test these delivery models in pragmatic trials, report fidelity and cost-effectiveness, and compare step-based versus intensity-guided prescriptions. Overall, wearables are best viewed as enabling components of comprehensive, structured and supportive care, rather than stand-alone solutions.

## Figures and Tables

**Table 1:** Summary of studies evaluating the use of wearable devices (e.g., fitness trackers, smartwatches, pedometers, accelerometers) to promote physical activity and improve clinical–metabolic health in adults with type 2 diabetes. Table including: study design, type and duration of activity monitoring, overall study duration, and main clinical outcomes. RCT: randomized controlled trial; HbA1c: glycated hemoglobin; IG: intervention group; CG: control group; BW: body weight; BMI: Body mass index; DBP: diastolic blood pressure; SBP: systolic blood pressure; PA: physical activity; CV: cardiovascular; HR: heart rate; TIR: time in range; FLI: fatty liver index; VAI: visceral adiposity index; HRQOL: health-related quality of life; AGEs: markers of advanced glycation; WHR: waist-to-height ratio; 6MWT: 6 min walking test; ABMP: Ambulatory Blood Pressure Monitoring.

Ref.	Author, Year of Publication (Country)	Study Type	Population/Sample	Type of Device Used for PA Monitoring	Overall Study Duration	Main Clinical Outcomes	Effect on HbA1c
[20]	Agboola et al., 2016 (USA)	Parallel-arm RCT	126 adults, 51.6% female	ActiPed+ (FitLinxx) pedometer	6 months	HbA1c, step counts, overall physical activity levels, device adherence	HbA1c decreased significantly within the IG. No significant differences between groups for HbA1c
[21]	Alghafri et al., 2018 (Oman)	Cluster RCT	232 adults, 59.1% female	Yamax Digi-Walker SW-200 pedometer, (Yamasa Tokei Keiki, Tokyo, Japan)	12 months	Weight, BMI, HbA1c, SBP/DBP, triglyceride levels	Significant within-group reduction in HbA1c in the intervention group (IG) No significant between-group difference in HbA1c
[22]	Alonso-Dominguez et al., 2019 (Spain)	RCT	204 adults, 45.6% female	Omron HJ-321 Triaxis pedometer + EVIDENT II smartphone app	12 months	Daily step count, aerobic steps, METs-min/week, sedentary time. BMI, waist circumference, postprandial glucose, lipid profile, SBP	No data on HbA1c reported
[23]	Arovah et al., 2018 (Indonesia)	Pilot RCT	43 adults, 62.8% female	Yamax SW-200 pedometer	24 weeks	Daily step count, HbA1c, fasting glucose, postprandial glucose	HbA1c decreased over time in both groups, with no significant differences between them.
[16]	Balducci et al., 2017 (Italy)	Cross-Sectional Study	300 adults, 38.7% female	MyWellness Key accelerometer (Technogym, Cesena, Italy)	7 days	Physical activity levels, sedentary time, HbA1c, fasting glucose, insulin resistance, BMI, waist circumference, fat mass, blood pressure, triglycerides, hs-CRP, and CV risk scores. Cardiorespiratory fitness, muscular strength, and flexibility	Higher PA associated with lower HbA1c; higher HbA1c predicted lower PA and sedentary time
[24]	Balducci et al., 2017 (Italy)	RCT	300 adults, 38.7% female	MyWellness Key accelerometer (Technogym, Cesena, Italy)	4 months	Physical activity levels, sedentary time, HbA1c, BP, lipids, renal function, BW, waist circumference, and inflammation	Significant HbA1c reduction in IG; greatest improvements observed with higher PA increase and sedentary time reduction
[25]	Balducci et al., 2022 (Italy)	RCT	300 adults, 38.7% female	MyWellness Key accelerometer (Technogym, Cesena, Italy)	3 years	Physical activity levels, sedentary time, VO_2_max, muscle strength, and flexibility, HbA1c, fasting glucose, BMI, waist circumference, triglycerides, SBP/DBP, high-sensitivity C-reactive protein. 10-year risk scores for coronary heart disease and stroke	Significant reduction in IG; extent of HbA1c improvement proportional to PA increase and sedentary time reduction
[10]	Cao et al., 2024 (UK)	Prospective Cohort Study	4003 adults, 37.0% female	Axivity AX3 triaxial accelerometer, (Newcastle upon Tyne, UK)	6.9 years	Physical activity levels, all-cause, cancer, and CV mortality	No data on HbA1c reported
[26]	Chlebowy et al., 2015 (USA)	Controlled Trial	62 adults, 64.5% female	MiniMitter^®^ accelerometer, (Respironics; Bend, OR, USA)	3 months	Physical activity adherence, Blood sugar levels, BMI, long-term blood sugar control, medication use	No significant between-group difference in long-term blood sugar control
[17]	Cirilli et al., 2019 (Italy)	Prospective observational	19 adults, 31.6% female	MyWellness Key gravitometer, (MyWellness Key; Technogym, Cesena, Italy)	3 months	Physical activity levels, antioxidant enzyme activities, oxidative stress markers, DNA damage, visceral-to-total fat ratio, SBP, endothelial function, pro-inflammatory microRNAs, angiogenic microRNAs, lipid profile	No data on HbA1c reported
[27]	Dahjio et al., 2016 (Cameroon)	Non-randomized interventional study (pre-post design)	23 adults, 100% female	NESTLE pedometer	12 weeks	Fasting blood glucose, body weight, BMI, waist circumference, visceral fat, VO_2_max, lean mass, overall fat mass, insulin sensitivity	No data on HbA1c reported
[28]	Fayehun et al., 2018 (Nigeria)	RCT (2 arms)	46 adults, 63% female	Digi-Walker SW-200 pedometer, (Yamax Inc., Tokyo, Japan)	11 weeks	Step count, HbA1c, BW, waist circumference, BP, HR	Significant HbA1c reduction in IG compared to CG
[15]	Gauthier et al., 2022 (France)	Observational, longitudinal, single-centre study	28 adults, 46% female	ActiGraph^®^ GT3X triaxal accelerometer	7 days	Physical activity levels, sleep duration, glycaemic variability, TIR	No data on HbA1c reported
[29]	Gu et al., 2020 (China)	2×2 factorial RCT	180 adults, 37.8% female	P084 SPORTWAY, pedometer + HBMP device, (P084, SPORTWAY, Shenzhen, China)	18 months	Physical activity levels, SBP/DBP, medication management	No data on HbA1c reported
[30]	Haxhi et al., 2024 (Italy)	RCT	267 adults, 39.3% female	MyWellness Key accelerometer, (Technogym, Cesena, Italy)	3 years	ALT, gamma-GT, FLI, hepatic steatosis index (HIS), AST, VAI, liver markers, VO_2_max, muscle strength, BMI, waist circumference	No data on HbA1c reported
[19]	Jiwani et al., 2020 (USA)	Clinical demonstration/pre-post interventional study	62 adults, 8% female	Pedometer (brand not specified)	12 months	BW, BMI, daily step count, walking speed, mobility, HbA1c	Significant HbA1c reduction at 3, 6, and 12 months post-intervention
[31]	Johnson et al., 2015 (Canada)	Controlled implementation trial	198 adults, 51% female	Yamax SW-200 pedometer	6 months	Daily step count, HbA1c, BW, waist circumference, BP, cholesterol, dietary intake (calories, glycemic index/load), HRQOL	No significant between-group difference in HbA1c
[8]	Kooiman et al., 2018 (Netherlands)	RCT	72 adults, 47.2% female	Fitbit Zip pedometer, (Fitbit Inc, San Francisco, CA, USA)	13 weeks	Physical activity levels, step count, HbA1c, BW, BMI, waist/hip ratio, AGEs	HbA1c improved in active IG subgroup; no overall between-group difference
[12]	L. P. de Oliveira et al., 2024 (Brazil)	RCT (2 arms)	35 adults, 48.6% female	HJ-321 Omron^®^ pedometer	16 weeks	Daily step count, 24 h, daytime, and nighttime SBP/DBP, BW, BMI, muscle mass, body fat, waist-to-hip ratio, glycemic control, lipid profile, insulin sensitivity	No significant between-group difference in glycemic control
[32]	Li et al., 2021 (China)	Multicenter RCT	101 adults, 23.8% female	Chest-worn HR band connected to R Plus Health app, (Recovery Plus Inc.),	3 months	Cardiopulmonary endurance, body fat%, HbA1c, muscle strength, HOMA-IR, cholesterol levels, WHR, BMI, medication management	Similar HbA1c reduction in both groups; no significant between-group difference
[14]	Masuda et al., 2021 (Japan)	Prospective Observational Cohort	94 adults, 28.7% female	TERUMO MT-KT02DZ accelerometer, (Tokyo, Japan)	6 months	Physical activity levels, daily step count, glycemic control, diabetes duration, incidence of complications and comorbidities	Higher step count associated with better glycemic control; poor control linked to low activity levels
[33]	Matsushita et al., 2022 (Japan)	RCT	29 adults, 13.8% female	Active Style Pro HJA-750C accelerometer, (OMRON Corporation, Kyoto, Japan)	12 weeks	Physical activity levels, HbA1c, daily step count, energy expenditure, lower extremity muscle strength, 6MWT performance, BMI, BP, lipid profile, body composition	Significant HbA1c reduction in IG compared to CG; correlated with increased step count
[7]	Miyauchi et al., 2016 (Japan)	RCT	187 adults, 32.6% female	MT-KT01, Terumo triaxial accelerometer, OR Modified MT-KT01 pedometer, (Tokyo, Japan)	6 months	Exercise adherence, HbA1c, BP, lipids, BMI, medication management	Greater HbA1c reduction at 2 months in activity monitor group; benefit sustained at 6 months in adherent participants
[13]	Paula et al., 2015 (Brazil)	RCT (2 arms)	40 adults, 55% female	Digi-Walker CW200 pedometer, (Yamax, Tokyo, Japan)	4 weeks	SBP, DBP (both office and 24 h ABPM), daily step count, dietary markers, urinary sodium and potassium, BNP, aldosterone, plasma renin activity, BMI, waist circumference, fat mass, fasting glucose, LDL cholesterol, triglycerides, HbA1c	Similar HbA1c reduction in both groups; not statistically significant
[34]	Rekha et al., 2020 (India)	RCT (2 arms)	34 adults, %female not specified	PINGKO Outdoor Multi-Function Pedometer	12 weeks	HbA1c, quality of life, BMI, body fat%, waist/hip ratio or BP	Significant HbA1c reduction in IG only
[35]	Sazlina et al., 2015 (Malaysia)	Three-arm RCT	69 adults, 46.4% female	Yamax Digi-Walker^®^ CW 700/701 pedometer, (Japan)	36 weeks	Physical activity levels, body fat%, cardiorespiratory fitness (6MWT), HbA1c, cardiovascular risk factors, body weight, BMI, waist circumference	No significant change in HbA1c across groups
[18]	Siddiqui et al., 2018 (South Africa)	Cross-sectional observational study	95 adults, 67.4% female	Multi-function pedometer (brand not specified)	4 months	BP, daily step count, HbA1c, BMI	HbA1c decreased in active group and increased in control; weak inverse association with step count
[36]	Tanaka et al., 2022 (Japan)	RCT (2 arms)	62 adults, 61.3% female	Lifecorder GS accelerometer, GS; Suzuken Co. Ltd. ver 2.20	6 months	Physical activity levels, HbA1c, BMI, self-management	No significant changes or group differences in HbA1c
[37]	Timurtas et al., 2022 (Turkey)	RCT (3 parallel groups)	75 adults, %female not specified	Smartphone (app) and wearable smartwatch (DIABETEX platform)	12 weeks	HbA1c, 6MWT distance, functional improvements	Greater mean HbA1c reduction in the supervised group, Modest, non-significant HbA1c changes in mobile app and smartwatch groups, Similar proportion of clinically meaningful improvement across groups
[9]	Yang et al., 2020 (USA)	Observational longitudinal study	60 adults, 72% female	Fitbit triaxial accelerometer and associated fitness app, (San Francisco, CA, USA)	6 months	Physical activity engagement levels, HbA1c, insulin use	Higher engagement linked to lower baseline HbA1c; no significant change across groups over time

## Data Availability

No new data were created or analyzed in this study.

## Abbreviations

The following abbreviations are used in this manuscript:

T2D	Type 2 diabetes mellitus
PA	Physical activity
DPP	Diabetes Prevention Programme
LPA	Light Physical Activity
MPA	Moderate Physical Activity
VPA	Vigorous Physical Activity
MVPA	Moderate-to-Vigorous Physical Activity
MET	Metabolic Equivalent of Task
WHO	World Health Organization
ADA	American Diabetes Association
HbA1c	Glycated Hemoglobin
VO_2_max	Maximal Oxygen Uptake
LDL	Low-Density Lipoprotein
ALT	Alanine Aminotransferase
γ-GT	Gamma-Glutamyl Transferase
NAFLD	Non-Alcoholic Fatty Liver Disease
BP	Blood Pressure
IDES	Italian Diabetes and Exercise Study
LMCMN	Lifestyle Medicine Case Manager Nurse
